# Different Faces of Hepatocellular Carcinoma as a Health Threat in 21st Century

**DOI:** 10.5812/hepatmon.9308

**Published:** 2013-02-04

**Authors:** Behzad Yeganeh, Mohammad Hashemi, Fredrick J. de Serres, Marek J. Los, Saeid Ghavami

**Affiliations:** 1 Department of Physiology, University of Manitoba, Winnipeg, Canada; 2 Cellular and Molecular Research Center, Zahedan University of Medical Sciences, Zahedan, IR Iran; 3 Department of Clinical Biochemistry, School of Medicine, Zahedan University of Medical Sciences, Zahedan, IR Iran; 4 Center for Evaluation of Risks to Human Reproduction, National Institute of Environmental Health Sciences, Research Triangle Park, Chapel Hill, USA; 5 Division of Cell Biology, Department of Clinical and Experimental Medicine, Integrative Regenerative Medicine Center (IGEN), Linköping University, Linköping, Sweden; 6 Manitoba Institute of Child Health, Winnipeg, Canada; 7 St. Boniface Research Centre, University of Manitoba, Winnipeg, Canada

**Keywords:** Biological Markers, Alpha 1 Antitrypsin, lobaplatin, Aflatoxins, Hepatitis B Virus, Hepatitis C

Hepatocellular carcinoma (HCC) is considered as the third and also one of the most frequent global cancers, accounting for more than half a million deaths annually (1, 2). There is a contentious rate in HCC incidence and mortality, which highlights the need for new and innovative therapeutic approaches. Over the last decade, the incidence of HCC remains the highest in Eastern Asia and Africa, while its frequency has grown progressively in western countries and in Japan as well (3). The molecular mechanisms of HCC-pathogenesis are not well-described, and the available information is less than that of other types of cancers such as breast and colon cancers. However in recent years, there have been major positive developments in HCC studies mainly because of improvements in clinical trials and the discovery of important genetic or epigenetic drivers of specific HCC subclasses, which caused development of more personalized treatments in HCC patients. Along with importance of HCC and high demands from clinicians and basic scientists, Hepatitis Monthly has compiled a special issue focusing on different aspects of HCC to cover the latest updates in this field. The rapid development in a vast array of techniques in various areas of cellular- and molecular cancer biology has led to major advances in understanding of the biology of HCC and other cancers. Therefore, specific molecular markers of biological behavior, diagnostic and prognostic significance that may also serve as a potential therapeutic target have gained significant interest (4). Abu El Makarem has discussed the latest information concerning HCC biomarkers in this issue (5). She has reviewed the importance of such HCC markers including alpha-fetoprotein (AFP) and des-gammacarboxy prothrombin (DCP), while underlying the major demand for developing more HCC biomarkers in order to decrease the mortality rate and increase the quality of the treatment in these patients. Lobaplatin (D-19466; 1, 2-diamminomethyl- cyclobutane-platinum (II)-lactate) is a new platinum derive from anti-tumor component that has shown some promises while testing in the lab. In recent years, the excellent safety profile with lower renal toxicity attracted marked attentions to this compound as a new chemotherapy agent as compared to cisplatin (6-8). It is currently used in a variety of tumor types such as human esophageal cancer, ovarian cancer, breast cancer, small cell lung cancer, colorectal carcinoma, and hepatocellular carcinoma (9, 10). In China, Lobaplatin has been approved for the treatment of chronic myelogenous leukemia (CML), metastatic breast cancer and small cell lung cancer (11). Recently, Wang et al. has provided evidence that Lobaplatin could also inhibit the proliferation of HCC cells with intact p53, via activation of p53 related pathways (11), thus it raised hopes that Lobaplatin will be added to the arsenal of anti-HCC-chemotherapeutics. 

Although chronic liver disease caused by hepatitis B virus (HBV) or hepatitis C virus (HCV) is the major risk factor for the development of HCC (12, 13), the fundamental mechanisms of their hepatocarcinogenesis are very diverse, partially due to differences between the biology of RNA viruses (with an integration capacity for the host genome) and DNA viruses (no genome integration capacity) (13). On the other hand, huge population is co-infected with human immunodeficiency virus (HIV) and HBV or HCV. In the United States, 70-90% of HIV-infected individuals have shown evidence of past or active infection with HBV (14). In 2009, it was estimated that of the 33.3 million HIV infected individuals all over the world, around five million have simultaneous HCV, and four million HBV co-infection (15-17). Zidan et al. have provided the latest epidemiological findings of HBV and HCV in Iran and then compared their results to those of Asia, Europe, United States, and Africa. They also discussed the incidence of HCC in these areas and correlate their data to HBV and HCV infection rates in each geographical area to highlight the importance of HBV and HCV infections in HCC incidence (18). Ng et al. have reviewed HBV and HCV related to HCC in the United States and have discussed the difference between HCV and HBV-originated HCC (19). The above papers not only highlight the problem and put it into a global context, but also they may help in the development of novel treatment strategies for each etiology of HCC while providing a more holistic view on the problem (19). Kew (20) has reviewed co-infections of HBV and HIV-1 in Sub-Saharan black Africans. He presents an interesting thesis which shows that the possible reasons of increasing the rate of HCC in recent years are the co-infection and thus a synergy between hepatocarcinogenec viruses and other viruses that might increase their carcinogenic potency (20). Liver transplantation is considered as one of the major therapeutic approaches in final stage or cirrhotic HCC patients (21). It is however limited to patients with minimum risk of tumor reappearance while they receive immunosuppressive therapy. The risk of recurrence is reduced in patients with favorable natural history and low alpha fetoprotein level (21). Du et al have considered the importance of proper anti-viral therapy in HCC patients before and after liver transplantation to decrease the chance of HCC recurrence in these patients (22). They concluded that anti-viral therapy, when carefully timed, could be a powerful tool in preventing HCC recurrence in these cases after liver transplantation, and would increase life quality after transplantation (22). 

Alpha 1-antitrypsin deficiency (AATD) is an inherited metabolic disorder in which mutations in the coding sequence of the serine protease inhibitor, prevent processing and proper folding of alpha 1-antitrypsin from endoplasmic reticulum and finally causes its hepatocyte accumulation (23-25). Alpha 1-antitrypsin deficiency is the most common genetic cause of liver disease in neonates and children (26, 27). The clinical presentation of liver disease in Z homozygote alpha 1-antitrypsin-deficient individuals is variable (9), however cirrhosis resulting from alpha 1-antitrypsin deficiency is an established risk factor of hepatocellular carcinoma (9). Thus, two manuscripts in the HCC-dedicated issue focus on the significance of AATD in HCC aethiology. Topic et al. have discussed the effect of different alpha-1 antitrypsin (AAT) deficient phenotpyes (ZZ, MZ, SZ) in HCC occurrence (28). They concluded that some specific AAT alleles and phenotypes could be responsible for HCC and that considering AATD is important in the area with high prevalence of AATD since early diagnosis of AATD would be beneficial to decrease the incidence of HCC. In another manuscript, Blanco et al. have investigated the frequency of AAT genes (Z and S) in 94 countries with informatics approach, namely: the ArcMap (29). This worldwide study provides extremely valuable information for all AATD related diseases including HCC and has significant impact in the field. 

S100A8 and S100A9 are two calcium and zinc binding proteins belonging to the S100-family, which could form homo- or hetero-dimer complex proteins and are highly expressed in neutrophils with potent antimicrobial properties (30, 31). Recent researches demonstrate that S100A8 and S100A9 are pro-inflammatory mediators released by cells of myeloid origin in response to cell damage, infection, or inflammation, and function as pro-inflammatory danger signals (30, 32, 33). It was recently revealed that S100A8 and S100A9 proteins increased the inflammatory cells infiltrating from precancerous lesions and HCC cells in Mdr2-/- mouse model (34). Interestingly, tumor-induced up-regulation of S100A8 and S100A9 proteins were found in myeloid precursor cells and have contributed to the recruitment and accumulation of myeloid-derived immune-suppressor cells at sites of tumor formation (34). Accordingly, in the compiled HCC volume, Maletzki and colleagues provided a comprehensive literature review highlighting the possible significance of S100A8, or S100A89 as HCC and colorectal carcinoma (mainly as diagnostic and prognostic markers). They concluded that it is too early to consider these proteins as the marker for specific cancers (35). 

Most components are processed, detoxified and metabolized in the liver. The toxic metabolites generated by these processes are the main causes of liver damage (36). Previous studies denoted that hepatocarcinogenesis is a long-term, multistage process in which multiple risk factors are involved (37). In the HCC volume, Uccelo et al. have focused on the importance of chemical exposure as a possible HCC risk factor (38). Their literature review includes a list of important chemicals that may be found at various occupational settings. They concluded that work place exposure to chemicals is a potential risk factor for HCC, however appropriate environmental studies should be arranged to reach a final conclusion for hepatotoxicity and hepatocarcinogenecity of different chemical compounds. 

Aflatoxins (AFs) are carcinogenic in several animal species but with variable potency. AFB1 is a major human hepatocarcinogen (39). Wu et al. have reviewed several manuscripts focusing on the epidemiological facts connecting nutritional revelation to AFB1 and risk of HCC, emphasizing on possible relations between AFB1 and HBV concerned in AFB1-related metabolism as well as DNA repair (40). They came to a very important conclusion, that dietary exposure to AFs is an important risk factor for HCC in Asia and sub-Saharan Africa region. 

Combined hepatocellular and cholangiocarcinoma (cHCC-CC) is a rare primary hepatic tumor that involves a mixture of both elements and its rate of reorganization is gradually increases (41, 42). It shares diverse features of both hepatocellular carcinoma (HCC) and cholangiocarcinoma (CC) (42), therefore its identifications and characterization are difficult and special caution should be given during the final diagnosis decision for this rare type of tumor (42). Al Hamoudi et al. have reported a cHCC-CC case and explained all important points that should be considered during the diagnosis of this tumor in the HCC special volume (43). Their manuscript will likely have a broad impact on the diagnosis of cHCC-CC. 

The imbalance between proliferation and cell death is a key feature observed in cancers including HCC (44). Phosphatase and tensin homolog deleted on chromosome ten (PTEN), mammalian target of rapamycin (mTOR), phosphatidylinositol 3’-kinase (PI3K) are key players that regulate cell proliferation and survival (44). Bassullu et al. have investigated the predictive and prognostic significance of c-erb-B2, EGFR, PTEN, mTOR, PI3K, p27, and ERCC1 expression in HCC in the Hepatitis Monthly HCC special issue (45), and concluded that deregulation of those pathways may serve as a prognostic marker for tumor aggressiveness. They also found that these markers have significant impact on prophecy of survival rate of HCC patients. 

Radiofrequency ablation (RFA) has been considered as a less invasive therapeutic treatment for HCC patients. In the past few years, RFA–based clinical protocols have accepted as an optional treatment strategy in many countries (46). In some ones, it is even being considered as first line treatment for HCC at early stage (46). Himoto et al have reviewed the latest status and progress in RFA as a therapy-tool the management of HCC (46). The HCC-dedicated volume of Hepatitis Monthly has covered the most important topics in the field, while attracting top experts in the relevant areas. The editors have concentrated their focus on compiling a volume that comprehensively reflects on the most important aspects of HCC-biology and therapy, and made an effort to include articles which may attract of both clinical- and basic scientists’ attention in the field. The editors are well-aware that still some important areas of the HCC-filed have not been discussed; however the time and space limitation prevented them from doing so. Those areas will certainly find proper coverage in the upcoming issues of Hepatitis Monthly.
